# Production of Indole-3-Acetic Acid and Degradation of 2,4-D by Yeasts Isolated from Pollinating Insects

**DOI:** 10.3390/microorganisms13071492

**Published:** 2025-06-26

**Authors:** Camila G. de Oliveira, Angela Alves dos Santos, Eduardo J. P. Pritsch, Stéfany K. Bressan, Anderson Giehl, Odinei Fogolari, Altemir J. Mossi, Helen Treichel, Sérgio L. Alves

**Affiliations:** 1 Laboratory of Yeast Biochemistry, Federal University of Fronteira Sul, Chapecó 89815-899, SC, Brazil; camila.girardi.oliveira@gmail.com (C.G.d.O.); angela.asds@gmail.com (A.A.d.S.); stefanybressan123@gmail.com (S.K.B.); andergiehl@gmail.com (A.G.); odinei.fogolari@uffs.edu.br (O.F.); 2 Laboratory of Microbiology and Bioprocesses, Federal University of Fronteira Sul, Erechim 99700-970, RS, Brazil; eduardo.pritsch@estudante.uffs.edu.br (E.J.P.P.); altemir.mossi@uffs.edu.br (A.J.M.)

**Keywords:** *Papiliotrema*, *Meyerozyma*, 2,4-dichlorophenoxyacetic acid, plant growth promotion, bioremediation, glyphosate, herbicide, bees, beetles

## Abstract

Synthetic herbicides such as glyphosate and 2,4-D are widely used in agriculture but can negatively impact non-target organisms, including microorganisms essential for ecological balance. Yeasts associated with pollinating insects play crucial roles in plant–insect interactions, yet their responses to herbicides remain understudied. This study aimed to evaluate the capacity of yeasts isolated from bees and beetles to produce indole-3-acetic acid (IAA), a plant-growth-promoting hormone, as well as their ability to tolerate or degrade glyphosate (in the commercial herbicide Zapp QI 620^®^) and 2,4-D (in the commercial Aminol 806^®^). Seven yeast strains were isolated from insects, identified via ITS sequencing, and assessed for IAA production in YPD medium. Growth assays were conducted under varying herbicide concentrations, and 2,4-D degradation was analyzed using high-performance liquid chromatography. All strains produced IAA, with *Papiliotrema siamensis* CHAP-239 exhibiting the highest yield (4.17 mg/L). Glyphosate completely inhibited growth in all strains, while 2,4-D showed dose-dependent effects, with four strains tolerating lower concentrations. Notably, *Meyerozyma caribbica* CHAP-248 degraded up to 46% of 2,4-D at 6.045 g/L. These findings highlight the ecological risks herbicides pose to beneficial yeasts and suggest the potential of certain strains for bioremediation in herbicide-contaminated environments. Overall, the study underscores the importance of preserving microbial biodiversity in the context of sustainable agriculture.

## 1. Introduction

The intensive use of synthetic herbicides is a widespread practice in modern conventional agriculture, playing a key role in weed control and crop productivity. Among the most commonly applied compounds are glyphosate (N-(phosphonomethyl)glycine) and 2,4-dichlorophenoxyacetic acid (2,4-D), with estimated global applications of 700,000 and 150,000 tons per year, respectively [1,2]. Despite their agronomic effectiveness, these herbicides have significant side effects, including adverse impacts on non-target organisms, especially microorganisms that are essential for ecological balance and soil health [3,4,5,6].

In this context, yeasts associated with flowers and pollinating insects represent a particularly relevant functional group. These microbes are involved in critical trophic and ecological interactions that support plant reproduction and pollinator health [7,8,9]. Through their fermentative activity, nectar-dwelling yeasts release volatile organic compounds (VOCs) that attract pollinators, facilitate microbial dispersal, and help maintain microbial community structure in both nectar and insect guts. However, environmental exposure to agrochemicals may compromise yeast viability, indirectly affecting pollination dynamics and, consequently, agricultural productivity [10,11].

Among the many ecological and biotechnological functions that such yeasts can perform, the production of indole-3-acetic acid (IAA) stands out. IAA is the most important auxin in plants, responsible for promoting apical and root development, enhancing nutrient uptake, and improving plant growth overall [12,13,14]. While IAA production has been extensively reported in plant-associated yeasts, it remains poorly explored in yeasts derived from pollinating insects. Investigating IAA synthesis in these microorganisms not only expands our understanding of their ecological roles but also reveals their potential as plant-growth-promoting bioinoculants in sustainable agriculture. Yeasts such as *Meyerozyma guilliermondii*, *Meyerozyma caribbica*, *Candida zemplinina*, *Rhodotorula mucilaginosa*, *Rhodotorula paludigenum*, *Carlosrosaea vrieseae*, and *Saccharomyces cerevisiae* have been recognized as IAA producers [13,15,16,17].

Simultaneously, evaluating the tolerance and/or degradation of herbicides by these yeasts is equally relevant. It is well documented that herbicides such as 2,4-D and glyphosate can induce oxidative stress in yeast cells, disrupt key metabolic pathways—such as the shikimate pathway—and inhibit cell viability even at agriculturally recommended concentrations [18,19,20,21,22]. On the other hand, some yeast species have demonstrated the ability to tolerate or even degrade these herbicides, suggesting promising applications in bioremediation [23,24,25,26,27].

In this study, we adopted an integrated approach to evaluate the biotechnological potential of yeasts isolated from bees and beetles. Our aim was to investigate the following: (i) their ability to produce IAA, (ii) their tolerance to the herbicides glyphosate and 2,4-D, and (iii) their capacity to degrade 2,4-D. To the best of our knowledge, this is the first study to assess these three traits in pollinator-associated yeasts, contributing to both the ecological understanding of these microorganisms and the development of biological strategies for herbicide-contaminated agroecosystems.

## 2. Materials and Methods

### 2.1. Isolation and Identification of Yeasts

Seven yeast strains were analyzed in this work (Table 1). Two of them had been reported in a previous study: *Meyerozyma caribbica* CHAP-242 and *M. caribbica* CHAP-248 were isolated from the stingless bee *Scaptotrigona postica* [28]. The following five strains are reported here for the first time: the strains CHAP-223, CHAP-224, CHAP-237, and CHAP-239 were isolated from the beetle *Astylus variegatus* (Melyridae; Coleoptera), and the strain CHAP-245 was isolated from the bee *Tetragonisca angustula* (Apidae; Hymenoptera). The insects were collected with an entomological aerial net during the Southern Hemisphere autumn of 2023, in Chapecó, SC, Brazil. The yeast strains were isolated as previously described [28]. For this study, the insects (bees and beetles) were submerged, separately, in a synthetic medium (6.7 g/L yeast nitrogen base, pH 5.0) with 10 g/L xylose and 0.2 g/L chloramphenicol (xylose was used here as the carbon source because it is the sugar routinely used for yeast isolation in our laboratory). Following previous studies described in the literature, the flasks containing the insects were incubated at 30 °C at 145 rpm until turbidity indicated growth [28,29,30,31]. Subsequently, 10 μL from each tube was streaked on agar plates containing the same medium described above, with the addition of 20 g/L agar. The yeast morphotypes were isolated and purified from these plates through repeated plating on the same solid medium.

For yeast species identification, the sequence of their Internal Transcribed Spacer (ITS1-5.8S rRNA-ITS2) was analyzed, amplified usg primers pair ITS1, 5′-GAACCWGCGGARGGATCA-3′, and ITS2, 5′-GCTGCGTTCTTCATCGATGC-3′ [32]. PCR was performed directly from the colony grown on the YNB medium, picked with a sterilized toothpick, as described by Tadioto et al. [29]. The amplified DNA was sequenced in the equipment MiSeq Sequencing System (Illumina Inc., San Diego, CA, USA). The consensus sequences, obtained from the forward and reverse reads, were edited and aligned using BioEdit software version 7.7 (https://bioedit.software.informer.com/, accessed on 20 June 2025). The sequences were compared with those from holotype strains in the NCBI Genbank using the basic local alignment search tool (BLAST, at http://www.ncbi.nlm.nih.gov, acessed on 20 June 2025), considering only hits with a query cover of ≥99%. After identification, the ITS regions of strains CHAP-223, CHAP-224, CHAP-237, CHAP-239, and CHAP-245 were deposited in NCBI (http://www.ncbi.nlm.nih.gov/, accessed on 20 June 2025), where they received the following numbers: PP554891, PQ788607, PP554956, PV544886, and PP554987, respectively.

### 2.2. Cellular Growth Assays and Indole-3-Acetic Acid Production

To evaluate cellular growth profiles, yeast strains were pre-grown in solid YPD medium (1% yeast extract, 2% peptone, 2% glucose, and 2% agar) for 48 h at 30 °C. After pre-cultivation, 1 µL of yeast cells was inoculated (using a 1 μL inoculation loop) into Erlenmeyer flasks filled to one-fifth of their functional capacity with liquid YPD medium (without agar). The cultures were kept under agitation and protected from light (to avoid indole-3-acetic acid degradation) on an orbital shaker at 145 rpm for 48 h at 30 °C. The inoculation of the strains into the media was considered time “0” (zero) and, from this moment on, samples were taken at 14, 24, 38, and 48 h of cultivation. Part of these samples was used to monitor cell growth by measuring optical density (OD) at 570 nm. In contrast, the other part was subjected to centrifugation (9000× *g*, 3 min), where the resulting supernatants were used for indole-3-acetic acid determination, as described below.

Indole-3-acetic acid (IAA) quantification was performed using a colorimetric method with Salkowski reagent (2 mL of 0.5 M Ferric Chloride III and 98 mL of 35% Perchloric Acid), following a protocol adapted from Fu et al. [33]. For this purpose, a standard curve with five different concentrations of IAA was initially prepared. In each analysis, 500 µL of Salkowski reagent was mixed with 500 µL of the sample. The reaction proceeded for 30 min at room temperature, protected from light. Subsequently, the samples were subjected to a spectrophotometer, and absorbance was recorded at 530 nm.

Statistical differences in IAA production by the strains were analyzed only at 48 h. For IAA values at this time, an analysis of variance (ANOVA) and Tukey’s test were applied to assess whether the differences between means were statistically significant. The results were presented with a 95% confidence interval (95% CI) using GraphPad Prism 8 software.

### 2.3. Cellular Growth Assays in the Presence of Synthetic Herbicides

To evaluate the impact of herbicides on yeast cell growth profiles, the same cell growth assays described above were performed in liquid YPD medium (as outlined in Section 2.2). However, in this case, the culture media were supplemented with different concentrations of synthetic herbicides. The commercial herbicides Zapp QI 620^®^, which contains glyphosate as its active ingredient, and Aminol 806^®^, which contains 2,4-D, were selected due to their widespread use in conventional agriculture in Brazil. Zapp QI 620^®^ contains 620 g/L of potassium glyphosate, 500 g/L of the glyphosate acid equivalent, and 740 g/L of other compounds. Aminol 806^®^ contains 806 g/L of 2,4-dichlorophenoxy acetate, 670 g/L of the 2,4-D acid equivalent, and 429 g/L of other ingredients.

These two products were used for the tests at four different concentrations: 25%, 50%, 100%, and 200% of the recommended dose specified on the product label. Based on these dilutions, the final concentrations of the active ingredients in the culture media were as follows: 3.255 g/L, 6.51 g/L, 13.02 g/L, and 26.04 g/L of glyphosate, and 1.51 g/L, 3.02 g/L, 6.045 g/L, and 12.09 g/L of 2,4-D. Culture supernatants were also sampled from these assays (via centrifugation at 9000× *g* for 3 min) for use in 2,4-D degradation analysis, as described below.

### 2.4. Analysis of 2,4-D Degradation

After 48 h of culture, samples were centrifuged, filtered (0.45 μm pore size, 13 mm diameter membrane filters), and diluted 15,000-fold for analysis via high-performance liquid chromatography (LCMS-2020, Shimadzu Corporation, Kyoto, Japan), using a C18 capillary column (NST18, 4,6 mm × 250 mm × 5 µm) under the following conditions: ionization source temperature of 250 °C; run time of 26 min; mobile phase consisting of ultrapure water, methanol, and formic acid; and a flow rate of 0.3 mL/min. The column oven temperature was maintained at 40 °C. The results, expressed using the mass detector, indicated the concentration of 2,4-D in the sample in mg/L.

## 3. Results

Two of the seven strains used in this study had been previously isolated and identified: *Meyerozyma caribbica* CHAP-242 and *M. caribbica* CHAP-248 [28]. The other five strains were isolated in this work from bees and beetles. The strains CHAP-223, CHAP-224, CHAP-237, and CHAP-239 were isolated from the beetle *A. variegatus*, and the strain CHAP-245 was isolated from the Brazilian native stingless bee *T. angustula*. The ITS regions of these strains were sequenced and compared to the holotype strains available in the GenBank database. The top three identity matches for each strain are displayed in Table 2. As a result, strain CHAP-223 was identified as *Papiliotrema rajasthanensis*, strain CHAP-245 as *Meyerozyma caribbica*, and strain CHAP-239 as *Papiliotrema siamensis*. For the isolates CHAP-224 and CHAP-237, assignment to a specific species was not possible due to identity values below 99%. However, they could be confidently assigned, respectively, to the genera *Tremella* and *Kurtzmaniella*, and are therefore referred to as *Tremella* sp. and *Kurtzmaniella* sp. (see Table 2). In particular, for CHAP-224, future studies may determine whether this strain represents a novel species, as its highest identity (with *T. shuangheensis*) did not exceed 95%.

**Figure 1:**
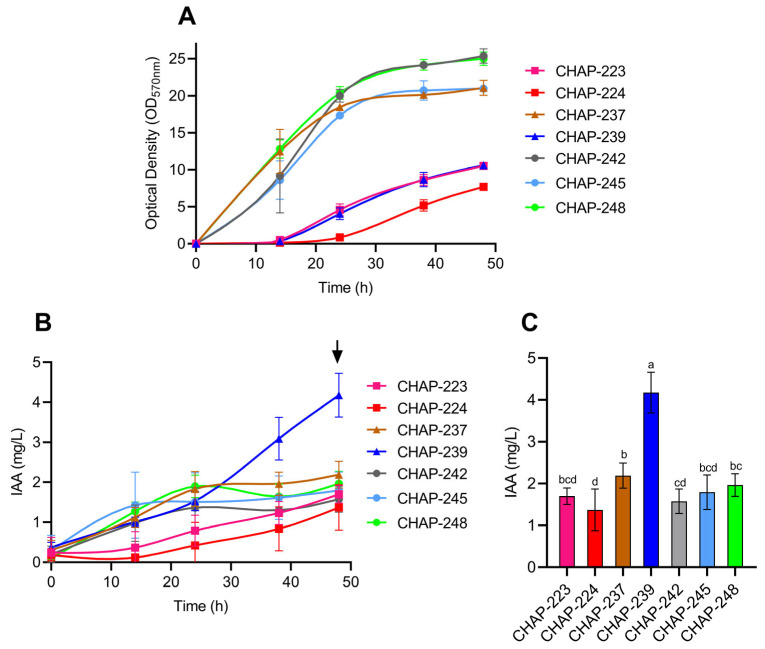
Yeast growth profiles and production of indole-3-acetic acid (IAA) over 48 h of cultivation in YPD medium: (**A**): Yeast growth profiles; (**B**): Production of IAA over 48 h; (**C**): IAA production at 48 h (indicated by the black arrow in (**B**)) and analysis of the statistical difference between productions in this point. Different letters represent significant differences (*p* ≤ 0.05). Data are presented as means of three independent experiments and their standard deviations.

In the present study, we selected indole-3-acetic acid (IAA) as a functional marker to evaluate the ecological and biotechnological potential of these yeasts. Accordingly, the seven strains were evaluated for their cell growth profiles and IAA production, as shown in Figure 1. All strains grew in YPD medium and produce IAA (Figure 1A,B). Notably, strains CHAP-237, CHAP-242, CHAP-245, and CHAP-248 exhibited faster growth and reached the highest optical density values after 48 h of culture (Figure 1A). In contrast, strain CHAP-239, which showed an extended lag phase and lower growth than the aforementioned strains, exhibited the highest IAA production after 48 h of incubation (Figure 1A,B). Identified as *Papiliotrema siamensis*, this strain produced 4.17 mg/L of IAA in 48 h of growth, significantly higher than the other six strains (Figure 1C). The remaining strains—*Papiliotrema rajasthanensis* CHAP-223, *Tremella* sp. CHAP-224, *Kurtzmaniella* sp. CHAP-237, and the three *Meyerozyma caribbica* strains CHAP-242, CHAP-245, and CHAP-248—produced 2.69, 1.37, 2.18, 1.57, 1.79, and 1.96 mg/L of this auxin, respectively (Figure 1C).

**Figure 2:**
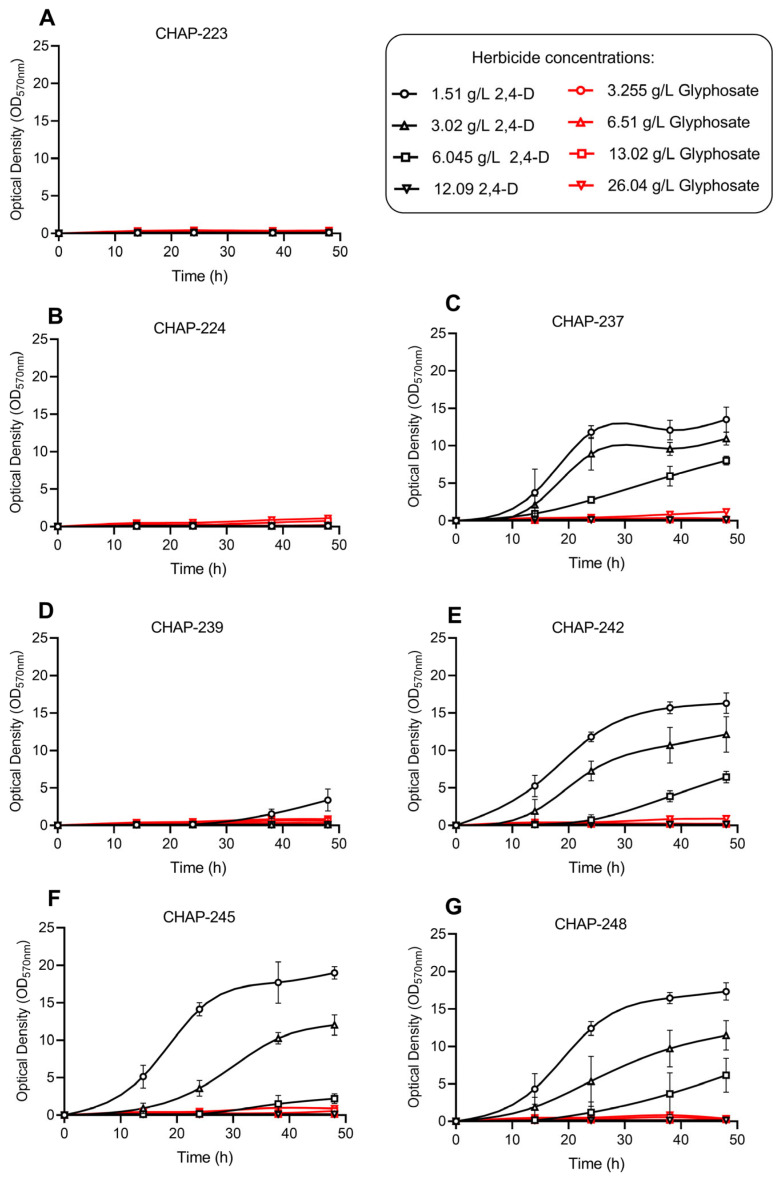
Yeast growth profiles under different concentrations of glyphosate (in the commercial herbicide Zapp QI 620^®^) and 2,4-D (in the commercial herbicid Aminol 806^®^): (**A**): *P. rajasthanensis* CHAP-223; (**B**): *Tremella* sp. CHAP-224; (**C**): *Kurtzmaniella* sp. CHAP-237; (**D**): *P. siamensis* CHAP-239; (**E**): *M. caribbica* CHAP-242; (**F**): *M. caribbica* CHAP-245; (**G**): *M. caribbica* CHAP-248.

We also evaluated the impact of different concentrations of the active ingredients glyphosate (in the commercial herbicide Zapp QI 620^®^) and 2,4-D (in the commercial Aminol 806^®^) on the cell growth of the isolated yeasts. The presence of synthetic herbicides in the culture medium negatively affected the growth of all strains, with the extent of inhibition varying by strain, active ingredient, and concentration of the herbicide added (Figure 2). No strain was able to grow in the presence of glyphosate, regardless of the concentration tested. Additionally, strains CHAP-223 and CHAP-224 were completely inhibited by all concentrations of 2,4-D (Figure 2A,B). Although strongly inhibited by 2,4-D, strain *P. siamensis* CHAP-239 could still grow when exposed to 1.51 g/L of this herbicide (Figure 2D); however, its growth reached only 66% of the level observed in herbicide-free YPD medium (Figure 1A). It is also worth noting that these three strains (CHAP-223, CHAP-224, and CHAP-239) exhibited longer lag phases even in the absence of herbicides (Figure 1A). For strains CHAP-223 and CHAP-239, this latency period was overcome after 15 h of cultivation, while CHAP-224 only entered exponential growth after 24 h.

The remaining four strains (*Kurtzmaniella* sp. CHAP-237, and the *M. caribbica* strains CHAP-242, CHAP-245, and CHAP-248) demonstrated different degrees of tolerance to 2,4-D, as shown in Figure 2C,E–G. The growth responses varied depending on the herbicide concentration in the medium. In general, growth inhibition was dose-dependent, with higher growth observed at lower concentrations of herbicide (1.51 and 3.02 g/L). In contrast, all four strains were severely affected at the highest concentration (12.09 g/L, twice the label-recommended dose), showing no growth during the 48 h incubation period. At the recommended dose of 6.045 g/L, the herbicide still inhibited growth, although to a lesser extent. The strains eventually entered an exponential phase, but this phase was poorly defined and extended throughout the evaluation period. At the end of the experiment with 6.045 g/L of 2,4-D, the percentage of cell growth inhibition was 90.08% for the strain *M. caribbica* CHAP-245 (Figure 2F), 76.05% for *M. caribbica* CHAP-242 (Figure 2E), 75.00% for *M. caribbica* CHAP-248 (Figure 2G), and only 58.59% for *P. siamensis* CHAP-237 (Figure 2C)—all compared to their respective growth in the absence of herbicide (Figure 1A).

Given the tolerance indicated above (in media with 1.51–6.045 g/L of 2,4-D), culture supernatants were analyzed using chromatography to assess the residual 2,4-D levels at the end of the cultivation period (Table 3). The results show that, at the lowest herbicide concentration (1.51 g/L), none of the yeasts degraded the herbicide. In contrast, in media with initial concentrations of 3.02 g/L or 6.045 g/L of 2,4-D, some strains were able to deplete up to 46% of this compound, as was the case with the strain *M. caribbica* CHAP-248 at 6.045 g/L. Under the same condition, *Kurtzmaniella* sp. CHAP-237 and the other *M. caribbica* strains (CHAP-242 and CHAP-245) degraded approximately 33%, 34%, and 30% of the initially available 2,4-D, respectively. Thus, our data suggest that lower growth inhibition does not necessarily correlate with greater herbicide degradation capacity.

## 4. Discussion

In this study, we tested seven yeast strains isolated from three different pollinating insects. Strains CHAP-242 and CHAP-248—isolated from the bee *S. postica*—had previously been sequenced and identified as *M. caribbica* and demonstrated strong capacity to produce 2-phenylethanol, an alcohol with broad biotechnological relevance [28]. Regarding the other five strains—isolated from the beetle *A. variegatus* and from the bee *T. angustula*—sequencing and ITS analysis revealed four distinct species: *Papiliotrema rajasthanensis* CHAP-223, *Tremella* sp. CHAP-224, *Kurtzmaniella* sp. CHAP-237, *Papiliotrema siamensis* CHAP-239, and *Meyerozyma caribbica* CHAP-245. Yeasts of the genera *Meyerozyma*, *Papiliotrema,* and *Kurtzmaniella* have previously been associated with insects [9,28,30,34,35,36]. In contrast, yeasts of the genus *Tremella* are more frequently isolated from decaying wood, leaves, plants, flowers, and soils [37,38], although they have also been recovered from the gut of beetles [39].

Among their ecological roles, yeasts can act as plant growth promoters, particularly by synthesizing bioactive substances such as indole-3-acetic acid (IAA), a key auxin involved in plant growth and development [12,13]. In microorganisms, IAA biosynthesis is generally thought to depend on tryptophan as a precursor [40]. However, not all microorganisms can synthesize this amino acid, mainly due to the high energetic cost of its biosynthesis [41]. Therefore, many microorganisms rely on their plant hosts or neighboring organisms for tryptophan, but its availability in natural environments is often limited [42]. In this context, some studies suggest that certain yeast species may also be capable of synthesizing IAA via alternative pathways that do not depend on exogenous tryptophan [13,42,43]. This metabolic flexibility could offer an ecological advantage to yeasts in nutrient-poor environments or, from a biotechnological perspective, enhance their potential as bioinoculants to support plant growth even under adverse conditions.

In our study, the seven yeast strains isolated from insects could produce IAA even without the addition of tryptophan to the culture medium. Six strains (one *Papiliotrema rajasthanensis*, one *Tremella* sp., one *Kurtzmaniella* sp., and three *Meyerozyma caribbica* strains) produced IAA concentrations between 1 and 2.5 mg/L after 48 h. Fernández-San Millán et al. [13] evaluated 69 yeast strains isolated from vineyards and found that 67 of them, including *M. caribbica* strains Mc-52 and Mc-57, could produce IAA under tryptophan-free conditions, reaching 2.08 and 1.06 mg/L, respectively. In their study, however, when tryptophan was added to the culture medium (at a concentration of 0.1%), IAA production increased to 4.11 and 2.22 mg/L, respectively, after a 7-day fermentation period [13]. Other yeasts have also been reported to produce IAA in the presence of 0.1% tryptophan, such as the soil isolate *Candida tropicalis* HY, which produced 2.6 mg/L of this auxin [44], and the Patagonian isolates *Candida saitoana* CRUB 1770 and *Saccharomyces eubayanus* CRUB 2014, which produced 1.58 and 0.18 mg/L of IAA, respectively [45]. Remarkably, our strain *P. siamensis* CHAP-239 produced 4.17 mg/L of IAA after only 48 h, even without tryptophan supplementation, highlighting its strong biotechnological potential for plant growth promotion.

In the plant–yeast relationship, tryptophan can be supplied to the microorganism by the plant, thus supporting microbial IAA synthesis and promoting mutualistic interactions [33,46]. In this context, a high capacity of microorganisms to convert tryptophan into IAA can benefit both organisms. For example, *Cyberlindnera saturnus*, an endophytic yeast, increased IAA production by 232% when supplemented with tryptophan and significantly enhanced maize root and shoot growth [47]. Likewise, *Carlosrosaea vrieseae* UFMG-CM-Y6724 produced 10 mg/L of IAA without tryptophan supplementation and 76.1 mg/L with it; filtrate-based formulations from this strain also promoted seedling development of the bromeliad *V. minarum* [17]. Based on these reports, it is likely that our strains would also produce higher IAA levels under tryptophan-enriched conditions, a hypothesis that should be tested in future work.

In agricultural systems, yeasts associated with plants and insects may be impacted by the toxic effects of herbicides, as exposure to these compounds can reduce their viability and consequently alter the dynamics of the biological interactions in which they are involved [11]. In this study, all seven strains were severely inhibited by glyphosate, showing no growth at any of the concentrations tested (3.255–26.04 g/L). Some yeasts, such as *Candida tropicalis* and *Cutaneotrichosporon cutaneum*, have shown to be able to grow in media with glyphosate to degrade it. However, it is essential to note that the concentration of glyphosate used in that study was 0.3 g/L, which is considerably lower than the concentrations tested in our assays. Nevertheless, similar to our results, in that study, even at low concentrations, glyphosate negatively affected yeast growth: exponential growth was observed only after 50 h for *C. cutaneum* and 100 h for *C. tropicalis* [23]. Similarly, this active ingredient also impaired the development of different *Saccharomyces cerevisiae* strains at concentrations ranging from 0.1% to 1.5% [48,49]. Thus, our results are consistent with previous findings and reinforce the notion that synthetic herbicides can strongly impact microbial communities that play critical roles in both agronomic and natural ecosystems.

When exposed to different concentrations of 2,4-D, four of the seven strains (identified as *M. caribbica* and *Kurtzmaniella* sp.) were able to grow. Although their tolerance varied, growth inhibition was clearly dose-dependent. The yeasts tolerated the lower concentrations of 2,4-D (1.51 and 3.02 g/L), but their growth was strongly impacted at higher levels. This pattern is similar to that described by Viegas et al. for *S. cerevisiae*, in which increasing 2,4-D concentrations prolonged the lag phase [18]. Likewise, Tavares et al. reported strong growth inhibition in *S. cerevisiae* even at low doses of 2,4-D (2.0, 4.0, and 6.0 µg/L) [50]. These findings recall earlier observations by Gilliam et al. who reported that bees fed with 2,4-D showed changes in the prevalence of intestinal yeasts compared to control colonies, suggesting that herbicide exposure may influence yeast abundance in pollinator-associated microbiomes [51]. Among our strains, *Kurtzmaniella* sp. CHAP-237 stood out as the most tolerant to the herbicide. Recently, Wang et al. found that a strain of *Kurtzmaniella* used in strawberry fermentation exhibited strong antioxidant activity, scavenging 2,2-diphenyl-1-picrylhydrazyl (DPPH) and 2,2′-azino-bis-(3-ethylbenzothiazoline-6-sulfonic acid) (ABTS) radicals [52]. One of the mechanisms by which 2,4-D affects yeast cells is through herbicide-induced oxidative stress [20,50]. Thus, the enhanced tolerance of strain CHAP-237 may be due to its antioxidant capacity, which could help neutralize the damaging effects of reactive oxygen species generated by 2,4-D exposure.

Nevertheless, this higher tolerance was not associated with enhanced herbicide degradation. Instead, the strain *M. caribbica* CHAP-248 was the most effective degrader of 2,4-D, despite being less resistant to this compound than the strain CHAP-237. Indeed, degradation and tolerance mechanisms can operate independently, as previously reported [11,20,53]. In this case, future research can assess the effectiveness of these yeasts in remediating environments contaminated with this compound. Additionally, further studies could aim to elucidate the molecular mechanisms that underlie both resistance to and degradation of 2,4-D.

## 5. Conclusions

This study presents an integrated assessment of three critical functional traits—IAA production, herbicide tolerance, and 2,4-D degradation—by wild yeasts isolated from pollinating insects. The production of indole-3-acetic acid (IAA), a key phytohormone associated with plant growth, was confirmed for all seven yeast strains evaluated. Notably, *Papiliotrema siamensis* CHAP-239 showed a high IAA yield (4.17 mg/L) even without the addition of tryptophan, highlighting its potential for use in plant growth promotion under nutrient-limited conditions.

In parallel, the impact of synthetic herbicides on yeast viability revealed that glyphosate—at agriculturally relevant concentrations—completely inhibited growth in all tested strains. The herbicide 2,4-D, although less toxic than glyphosate, significantly impaired growth in a dose-dependent manner, with only four strains (from the genera *Meyerozyma* and *Kurtzmaniella*) showing any level of tolerance.

Importantly, those tolerant strains also demonstrated varying abilities to degrade 2,4-D, with *M. caribbica* CHAP-248 achieving up to 46% degradation at 6.045 g/L of active ingredient. These findings demonstrate that tolerance and degradation are not necessarily correlated and may involve distinct physiological mechanisms.

Taken together, our results emphasize the vulnerability of beneficial yeasts—particularly those involved in insect–plant–microbe interactions—to herbicide exposure. At the same time, they highlight the potential of certain strains to act as bioinoculants and bioremediation agents. By combining these three perspectives, this work contributes to a broader understanding of how pollinator-associated yeasts can support both ecological resilience and sustainable agricultural practices.

## Figures and Tables

**Table 1:** Yeast strains utilized in this study.

Strains	Source of Isolation	Reference
CHAP-223	Angora beetle (species *Astylus variegatus*)	This study
CHAP-224	Angora beetle (species *Astylus variegatus*)	This study
CHAP-237	Angora beetle (species *Astylus variegatus*)	This study
CHAP-239	Angora beetle (species *Astylus variegatus*)	This study
CHAP-242	Stingless bees (species *Scaptotrigona postica*)	Fenner et al. [28]
CHAP-245	Stingless bees (species *Tetragonisca angustula*)	This study
CHAP-248	Stingless bees (species *Scaptotrigona postica*)	Fenner et al. [28]

**Table 2:** Percent identity matrix of ITS regions between the newly isolated CHAP strains and their closest related species, considering a query cover of ≥99% *.

Strains	Percent Identity Relative to:		
	*Papiliotrema rajasthanensis*	*Papiliotrema odontotermitis*	*Papiliotrema laurentii*
CHAP-223	99.05%	96.39%	95.63%
	*Tremella shuangheensis*	*Teunia tronadorensis*	*Teunia globosa*
CHAP-224	94.27%	86.71%	85.99%
	*Kurtzmaniella quercitrusa*	*Kurtzmaniella hittingeri*	*Danielozyma litseae*
CHAP-237	98.01%	94.66%	93.20%
	*Papiliotrema siamensis*	*Papiliotrema perniciosa*	*Papiliotrema nemorosa*
CHAP-239	99.34%	98.03%	96.71%
	*Meyerozyma caribbica*	*Meyerozyma carpophila*	*Meyerozyma guilliermondii*
CHAP-245	99.66%	99.49%	98.99%

* NCBI accession numbers of the sequences used to assemble this table: *P. rajasthanensis* (NR_155678.1), *P. odontotermitis* (NR_156605.1), *P. laurentii* (KY104469.1), *T. shuangheensis* (MK050285.1), *T. tronadorensis* (MF959620.1), *T. globosa* (MK050288.1), *K. quercitrusa* (NR_163508.1), *K. hittingeri* (NR_185511.1), *D. litseae* (KU570384.1), *P. siamensis* (NR_155608.1), *P. perniciosa* (NR_137653.1), *P. nemorosa* (KY104472.1), *M. caribbica* (MH545919.1), *M. carpophila* (MK394110.1), and *M. guilliermondii* (MH545918.1).

**Table 3:** Residual concentration of 2,4-D after culturing the strains CHAP-237, CHAP-242, CHAP-245, and CHAP-248 in media containing 1.51, 3.02, and 6.045 g/L of this herbicide. Values (in g/L) are presented as average ± standard deviation of two independent experiments.

	Residual Concentration of 2,4-D in Cultures That Started with:		
Strains	1.51 g/L	3.02 g/L	6.045 g/L
*Kurtzmaniella* sp. CHAP-237	1.48 ± 0.46	1.96 ± 0.07	4.05 ± 0.34
*Meyerozyma caribbica* CHAP-242	1.46 ± 0.01	2.98 ± 0.14	3.99 ± 0.95
*Meyerozyma caribbica* CHAP-245	1.65 ± 0.13	1.80 ± 0.04	4.20 ± 0.23
*Meyerozyma caribbica* CHAP-248	1.50 ± 0.53	1.88 ± 0.20	3.24 ± 0.30

## Data Availability

The original contributions presented in this study are included in the article. Further inquiries can be directed to the corresponding authors.
